# Case Report: Pediatric isavuconazole supratherapeutic exposure: a case of long-term use, CYP3A5 poor metabolizer genotype and concomitant CYP3A4 medications

**DOI:** 10.3389/fphar.2026.1807350

**Published:** 2026-03-23

**Authors:** Manman Liu, Lan Li, Chen Zhou, Changying Luo, Xiaodong Wang, Juan Wu

**Affiliations:** 1 Department of Pharmacy Administration, Shanghai Children’s Medical Center, School of Medicine, Shanghai JiaoTong University, Shanghai, China; 2 Blood and Marrow Transplantation Center, Shanghai Children’s Medical Center, School of Medicine, Shanghai JiaoTong University, Shanghai, China

**Keywords:** isavuconazole, pediatric, therapeutic drug monitoring, CYP3A5, long-term therapy, drug-drug interaction

## Abstract

Isavuconazole has high oral bioavailability and a predictable pharmacokinetic profile, and is a first-line agent for invasive fungal disease in adults. In children, its use is off-label. A 14-year-old boy received oral isavuconazole (200 mg daily) for pulmonary aspergillosis after allogeneic hematopoietic stem cell transplantation. After 2 months of therapy, the steady-state trough concentration increased from 2.2 mg/L to 15.0 mg/L, well above the suggested range (2.0–5.0 mg/L). Pharmacogenetic analysis revealed a CYP3A5∗3/∗3 (poor metabolizer) genotype. The drug accumulation likely reflected a combined effect of no CYP3A5 activity, long-term therapy, and possible competition within the CYP3A4 pathway from concomitant medications. It is still unclear whether these high levels cause toxic reactions. This case highlights the need for baseline and follow up steady-state therapeutic drug levels in pediatric patients receiving off-label isavuconazole, especially during extended therapy. Monitoring may help detect supratherapeutic exposure early and guide dose adjustments to optimize the therapeutic outcomes.

## Introduction

Invasive fungal disease (IFD) is a major cause of morbidity and mortality after allogeneic hematopoietic cell transplantation (HCT) (Groll et al., 2021). The latest European Conference on Infections in Leukemia (ECIL) guidelines recommended isavuconazole as a first-line agent for invasive aspergillosis in adult HCT recipients because it is better tolerated than voriconazole (Tissot et al., 2017). Pediatric data are still limited, but the guideline panel has provisionally extended this recommendation to children (Groll et al., 2021). As a result, off-label use in pediatric patients is becoming more common.

Isavuconazole is a broad-spectrum triazole that was approved in 2015. It has near-complete oral bioavailability, linear pharmacokinetics, and relatively low variability between patients (Ellsworth and Ostrosky-Zeichner, 2020). Based on these features, major guidelines, including ECIL and the Infectious Diseases Society of America (IDSA), do not recommend routine therapeutic drug monitoring (TDM) (Patterson et al., 2016). However, this advice mainly comes from short-term studies in hospitalized adults and may not apply to all patient groups. Emerging evidence suggests that some populations, including pediatric patients and those on long-term therapy, may have unpredictable drug exposure and may benefit from TDM (Borman et al., 2020; Gatti et al., 2025).

We report a pediatric case of marked isavuconazole accumulation during long-term oral therapy for pulmonary aspergillosis after allogeneic HCT. This report discusses how long-term administration and a CYP3A5 poor metabolizer genotype may have contributed to supratherapeutic exposure, highlighting a situation not well addressed by current dosing practice.

## Case/treatment

This is a 14-year-old boy who received allogeneic HCT following a diagnosis of primary immunodeficiency at the age of 9. The post-transplant course was complicated by graft-versus-host disease (GVHD), severe gastrointestinal bleeding with hypovolemic shock, cytomegalovirus (CMV)/Epstein-Barr virus (EBV) infection, and hemorrhagic cystitis. These conditions were managed with supportive care and a series of immunosuppressive therapies over the subsequent 5 years, including methylprednisolone, cyclosporine, sirolimus, tacrolimus, ruxolitinib, mycophenolate mofetil, and besudil mesylate.

**FIGURE 1:**
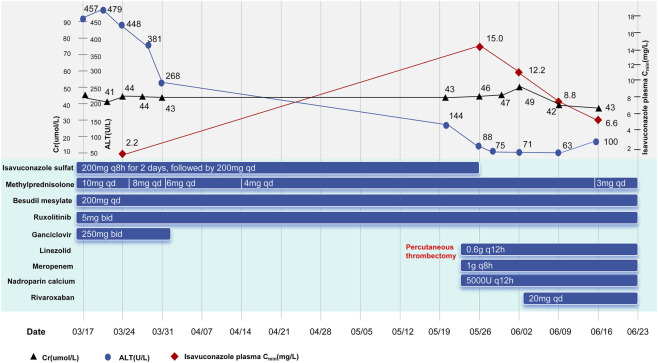
Main drug treatment process and laboratory tests.

In January 2025, the patient (153.0cm, 54.8 kg) was diagnosed with probable pulmonary IFD. The diagnosis was prompted by the development of new respiratory symptoms, including a productive cough and dyspnea. CT scan of the chest revealed multiple patchy infiltrates and mild bronchiectasis in both lungs. Microbiological evidence was obtained from a positive bronchoalveolar lavage fluid (BALF) galactomannan antigen test. This finding was further supported by next-generation sequencing (NGS) results revealing the presence of *Aspergillus fumigatus* and *Aspergillus flavus* in BALF. Due to elevated transaminase levels resulting from treatment with voriconazole and posaconazole, despite TDM results being within the target range, oral isavuconazole sulfate was initiated in March using a loading dose of 200 mg every 8 h for 2 days, followed by a once-daily maintenance regimen. At the time of isavuconazole initiation, the patient was being maintained on methylprednisolone, ruxolitinib, and besudil mesylate for chronic GVHD management, alongside ganciclovir for CMV infection. Detailed doses and timelines were shown in Figure 1.

TDM of isavuconazole 8 days after initiation revealed a steady-state trough concentration (C_min_) of 2.2 mg/L, within the therapeutic range (2.0–5.0 mg/L) (Gilbert et al., 2025). All C_min_ s in our institution were obtained by drawing blood samples 0.5 h prior to the scheduled daily dose.

In May 2025, the patient developed deep vein thrombosis (DVT) in the right lower extremity, for which he underwent percutaneous thrombectomy, followed by oral rivaroxaban for anticoagulation. A repeat TDM 1 week later revealed a supratherapeutic isavuconazole plasma C_min_ of 15.0 mg/L. In response to this significant drug accumulation and considering the concurrent improvement in pulmonary symptoms, isavuconazole was discontinued. Subsequent monitoring showed a gradual decline in plasma C_min_, measuring 12.2 mg/L, 8.8 mg/L, and 6.6 mg/L at 7, 14, and 21 days after discontinuation, respectively. Following the washout of the drug, antifungal therapy was not reinitiated. This approach was supported by sustained clinical and radiological improvement, including the resolution of respiratory symptoms and the gradual regression of nodular lesions on follow-up chest CT imaging, along with normalization of galactomannan antigen level. The patient remained free of any clinical or radiological signs of relapse throughout the subsequent follow-up period. A retrospective review of the patient’s whole-exome sequencing data, which had been obtained during the diagnostic workup for the primary disease prior to transplantation, revealed homozygous loss-of-function alleles consistent with a poor metabolizer status for both CYP3A5 (*3/*3) and UGT1A1 (*28/*28), in contrast to a CYP3A4 genotype indicative of a normal metabolizer phenotype. Based on the three post-discontinuation plasma concentrations, the estimated apparent terminal half-life (t_1/2_) of isavuconazole in this patient was calculated to be approximately 385 h. This value is substantially longer than the mean t_1/2_ of approximately 110 h reported. There were no adverse reactions in laboratory parameters or clinical manifestations associated with the administration of isavuconazole.

## Discussion

In this case, the patient was initially treated with voriconazole but switched to posaconazole due to elevated transaminases. When transaminase levels failed to improve, isavuconazole was initiated. Previous studies indicate that isavuconazole exhibits similar antifungal activity against *Aspergillus* species as voriconazole, and superior efficacy compared to posaconazole (Gilbert et al., 2025). Clinical evidences show that isavuconazole has a more favorable safety profile: the drug-related discontinuation rate is significantly lower than voriconazole (8% vs. 14%) (Maertens et al., 2016), and the incidence of drug-induced liver injury (1.22%) is much lower than that of voriconazole (4.58%) and posaconazole (3.99%) (Zhou et al., 2022). In this case, despite a significant increase in isavuconazole C_min_ (up to 15.0 mg/L), the ALT levels decreased instead, further supporting its relatively favorable hepatic safety profile.

This pediatric patient, with height and weight approaching adult parameters, received a standard adult dosing regimen of isavuconazole. The initial TDM result confirmed a steady-state C_min_ of 2.2 mg/L. While the optimal therapeutic range continues to be refined, maintaining C_min_s between 2.0 mg/L and approximately 5.0 mg/L is generally supported to optimize efficacy while minimizing the risk of toxicity (Tan et al., 2025). This result supports using adult doses in pediatric patients of comparable body size to achieve target exposure initially. However, the patient subsequently developed a DVT in the right lower extremity after 2 months of continuous therapy. To date, no thromboembolic events have been attributed to isavuconazole in licensing studies or on post marketing surveillance (Maertens et al., 2018; Marty et al., 2016). This DVT may be due to the patient’s underlying risk factors, including a larger body habitus and prolonged periods of bed rest, which are well-established risks for venous thromboembolism.

The patient’s C_min_ of isavuconazole increased from 2.2 mg/L to 15.0 mg/L after 2 months of continuous therapy, exceeding the recommended upper limit. This marked accumulation may be explained by several factors. First, isavuconazole has a long elimination t_1/2_ (approximately 110 h), so sustained administration can lead to drug accumulation, as reported in previous studies (Cojutti et al., 2021). Second, pharmacogenetic analysis based on whole-exome sequencing data revealed that the patient was a CYP3A5 poor metabolizer (CYP3A5*3/*3). This genotype is well-established to result in a defective enzyme, which significantly impairs the metabolism of various drugs. Indeed, there is significant geographical variation in the frequency of the CYP3A5*3 variant. It is approximately 50% in African Americans, 70% in Chinese, and as high as 90% in Caucasians (Zhou et al., 2009). Given that both preclinical studies and case reports indicate that CYP3A4 and CYP3A5 are the primary isoenzymes responsible for the metabolism of isavuconazole (Townsend et al., 2017; Van Daele et al., 2021; Soman et al., 2025), the patient’s CYP3A5*3/*3 genotype may similarly explain the slowed elimination observed in this case. This association is consistent with findings for other medications, such as tacrolimus and cyclosporine A (Dung et al., 2025; Su et al., 2019). Additionally, drug-drug interactions (DDIs) may have played a role. Concomitant medications administered to the patient, including ruxolitinib, and besudil mesylate, are substrates of CYP3A4 (Appeldoorn et al., 2023; Grymonprez et al., 2023). These drugs may compete for this pathway and reduce the metabolic clearance of isavuconazole. As noted in the results, the patient’s estimated apparent terminal t_1/2_ of approximately 385 h is notably longer than the widely cited mean of 110 h. This finding suggests that, beyond competitive inhibition, other mechanisms such as a potential saturation of elimination pathways in this specific patient might have contributed to the prolonged t_1/2_. In summary, the patient’s CYP3A5 poor metabolizer genotype, and concomitant use of CYP3A4 substrates likely impaired isavuconazole clearance. This impairment, compounded by long-term dosing, appears to have driven metabolic pathway saturation, as reflected by the markedly prolonged t_1/2_ and the consequent supratherapeutic concentration.

UGTs are an important family of Phase II metabolizing enzymes. In this patient, whole-exome sequencing also revealed a UGT1A1*28 genotype (*28/*28), which is known to potentially cause severe cumulative toxicity with irinotecan (Geng et al., 2025). However, no association has been reported to date between this genotype and isavuconazole, and the relationship requires further investigation.

Whether elevated drug levels lead to toxicity remains unclear. A study by Emily et al., after adjusting for age and indication, found that a one-unit (1 mg/L) increase in isavuconazole serum concentration was significantly associated with a 26% increase in the linear hazard ratio for experiencing an adverse event (P = 0.0254) (Huang et al., 2025). However, in this case, the association between the DVT in the lower limb and the excessively high isavuconazole concentration remains unclear. Notably, no other adverse reactions, such as abnormalities in laboratory parameters or clinical manifestations, were observed.

This report has several limitations that warrant consideration. First, as a retrospective case report of a single patient, our findings cannot establish causality or be generalized to the broader population. The central hypothesis regarding the role of the CYP3A53/*3* genotype, while biologically plausible and supported by prior case reports, remains speculative. Second, the TDM was conducted based on clinical discretion rather than a predefined protocol. We did not perform TDM measurements between the initial assessment and the discovery of supratherapeutic levels, which obscures the precise trajectory of drug accumulation. Third, the lack of TDM for co-administered medications (e.g., ruxolitinib and besudil), due to resource constraints, prevents definitive conclusions regarding potential DDIs. To address these limitations, future research will need to establish a large-scale database to develop population pharmacokinetic (PopPK) or physiologically based pharmacokinetic (PBPK) models for isavuconazole. These models may enable quantitative dissection of the interplay between genetic polymorphisms and DDIs on isavuconazole exposure and systemic pharmacodynamics.

## Conclusion

TDM may be a valuable tool in pediatric patients receiving isavuconazole, particularly in the context of pharmacogenetic differences and complex polypharmacy. Given the potential for variable drug exposure, TDM could help guide individualized dosing strategies and optimize therapeutic outcomes during long-term treatment.

## Data Availability

The original contributions presented in the study are included in the article/supplementary material, further inquiries can be directed to the corresponding authors.
